# Physical Activity Rewires the Human Brain against Neurodegeneration

**DOI:** 10.3390/ijms23116223

**Published:** 2022-06-02

**Authors:** Jose A. Santiago, James P. Quinn, Judith A. Potashkin

**Affiliations:** 1 NeuroHub Analytics, LLC, Chicago, IL 60605, USA; jose.santiago.ecm@gmail.com; 2 Q Regulating Systems, LLC, Gurnee, IL 60031, USA; jim.quinn@qregulatingsystems.com; 3 Center for Neurodegenerative Diseases and Therapeutics, Cellular and Molecular Pharmacology Department, The Chicago Medical School, Rosalind Franklin University of Medicine and Science, North Chicago, IL 60064, USA

**Keywords:** Alzheimer’s disease, frontotemporal dementia, Huntington’s disease, Parkinson’s disease, physical activity

## Abstract

Physical activity may offset cognitive decline and dementia, but the molecular mechanisms by which it promotes neuroprotection remain elusive. In the absence of disease-modifying therapies, understanding the molecular effects of physical activity in the brain may be useful for identifying novel targets for disease management. Here we employed several bioinformatic methods to dissect the molecular underpinnings of physical activity in brain health. Network analysis identified ‘switch genes’ associated with drastic hippocampal transcriptional changes in aged cognitively intact individuals. Switch genes are key genes associated with dramatic transcriptional changes and thus may play a fundamental role in disease pathogenesis. Switch genes are associated with protein processing pathways and the metabolic control of glucose, lipids, and fatty acids. Correlation analysis showed that transcriptional patterns associated with physical activity significantly overlapped and negatively correlated with those of neurodegenerative diseases. Functional analysis revealed that physical activity might confer neuroprotection in Alzheimer’s (AD), Parkinson’s (PD), and Huntington’s (HD) diseases via the upregulation of synaptic signaling pathways. In contrast, in frontotemporal dementia (FTD) its effects are mediated by restoring mitochondrial function and energy precursors. Additionally, physical activity is associated with the downregulation of genes involved in inflammation in AD, neurogenesis in FTD, regulation of growth and transcriptional repression in PD, and glial cell differentiation in HD. Collectively, these findings suggest that physical activity directs transcriptional changes in the brain through different pathways across the broad spectrum of neurodegenerative diseases. These results provide new evidence on the unique and shared mechanisms between physical activity and neurodegenerative diseases.

## 1. Introduction

Physical activity has been recognized as a lifestyle modification, beneficial across multiple domains, including cardiovascular disease, the immune system, and brain health. Epidemiological studies have shown that physical activity counteracts the effect of aging and reduces the risk of Alzheimer’s disease (AD) and related dementias [1,2]. Daily physical activity, including walking, is associated with cognitive improvement in older adults [3,4]. While the benefits of physical activity in mental health are supported by most epidemiological studies [2,5,6], the molecular mechanisms by which it improves cognition and confers neuroprotection remain largely unknown. Although the effects of physical activity have been reported in AD previously [7], investigations at the molecular level in other common neurodegenerative diseases, including Parkinson’s disease (PD), Huntington’s disease (HD), and frontotemporal dementia (FTD), are lacking. Given the high prevalence of neurodegenerative diseases and the limited therapeutics available, research on non-pharmacological interventions, such as physical activity and diet, is expected to provide an alternative option for disease management and prevention.

Previous studies have identified gene signatures and biological pathways associated with physical activity, aging, and neurodegeneration. For example, differentially expressed genes in the hippocampus of AD and aging individuals significantly overlapped with those of physically active subjects [7]. Another study identified neurogenesis and the negative regulation of T cell-mediated inflammation as the most overrepresented pathways linked to high physical activity [8]. Recently, lower microglial activation has been suggested as a potential mechanism linking physical activity and age-related brain health in humans [9]. These studies used transcriptomic data from participants enrolled in the Rush Memory and Aging Project (MAP), a cohort study that evaluated lifestyle factors and chronic conditions associated with aging, cognitive decline, and AD longitudinally for up to 18 years or until death [10].

This study builds on previous work and employs several bioinformatic approaches to decipher the connection between physical activity and brain health. Among the bioinformatic methods, we used Switch Miner software to build co-expression networks to identify ‘switch genes’; genes associated with drastic transcriptional changes in the hippocampus of aged and cognitively intact subjects who engaged in different levels of physical activity. This analysis has identified switch genes and mechanisms of disease for several neurodegenerative diseases, including AD, FTD, and amyotrophic lateral sclerosis (ALS) [11,12,13]. Additionally, we compared the transcriptome of physically active subjects with those of neurodegenerative diseases to identify shared and unique pathways affected by physical activity. We showed that gene expression patterns associated with physical activity opposed those of neurodegenerative diseases. Further, we observed that physical activity might promote neuroprotection through different pathways across the spectrum of neurodegenerative diseases.

## 2. Results

### 2.1. Database Mining for Transcriptomic Studies

**Figure 1:**
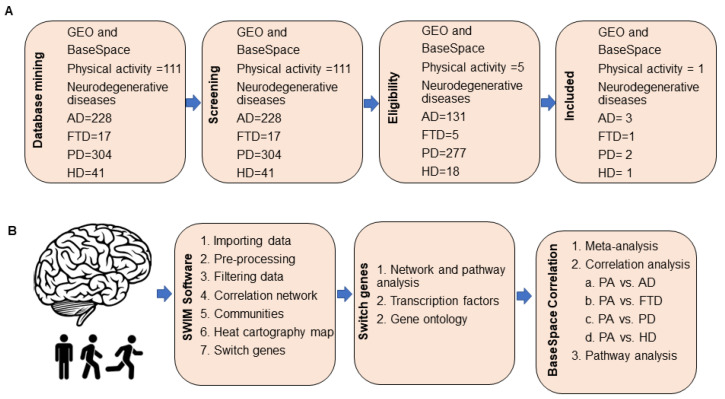
Overall study design. (**A**) We searched GEO and BaseSpace Correlation Engine (BSCE) databases for brain transcriptomic studies from subjects who engaged in different levels of physical activity and individuals with neurodegenerative diseases, including Alzheimer’s (AD), Parkinson’s (PD), Huntington’s diseases (HD), and frontotemporal dementia (FTD). Studies that met our inclusion/exclusion criteria were analyzed further. (**B**) Dataset GSE110298 containing brain transcriptomic data from aged cognitively intact individuals who engaged in different physical activity levels were analyzed by SWIM software to identify switch genes. Pathway and transcription factor analyses were performed in NetworkAnalyst. Gene ontology associations of switch genes were retrieved from HUGO database. Transcriptomic hippocampal data from subjects engaged in high and moderate physical activity were compared to those engaged in low physical activity using the meta-analysis and correlation engine tool in BSCE. Using this tool, we compared the transcriptome of physically active subjects with patients with neurodegenerative diseases to identify shared and unique pathways affected by physical activity.

We searched the Gene Expression Omnibus (GEO) and BaseSpace Correlation Engine (BSCE, Illumina, Inc., San Diego, CA, USA) databases to identify microarrays from aged cognitively intact individuals who engaged in physical activity and subjects with neurodegenerative diseases. The database search retrieved the following arrays: physical activity, GEO = 106 and BSCE = 5; neurodegenerative diseases: GEO (AD = 131, PD = 277, HD = 23, FTD = 5), BSCE (AD = 97, PD = 77, FTD = 12, HD = 18). One array containing transcriptomic and physical activity data from elderly individuals with unimpaired cognition (GSE110298) and seven microarrays containing transcriptomic data from patients with neurodegenerative diseases, including AD, PD, FTD, and HD, met our inclusion/exclusion criteria and were analyzed further (See Section 4). The overall workflow strategy is presented in Figure 1. The list of microarrays analyzed in this study is provided in Table S1.

### 2.2. Identification of Switch Genes in the Hippocampus of Physically Active Subjects

**Figure 2:**
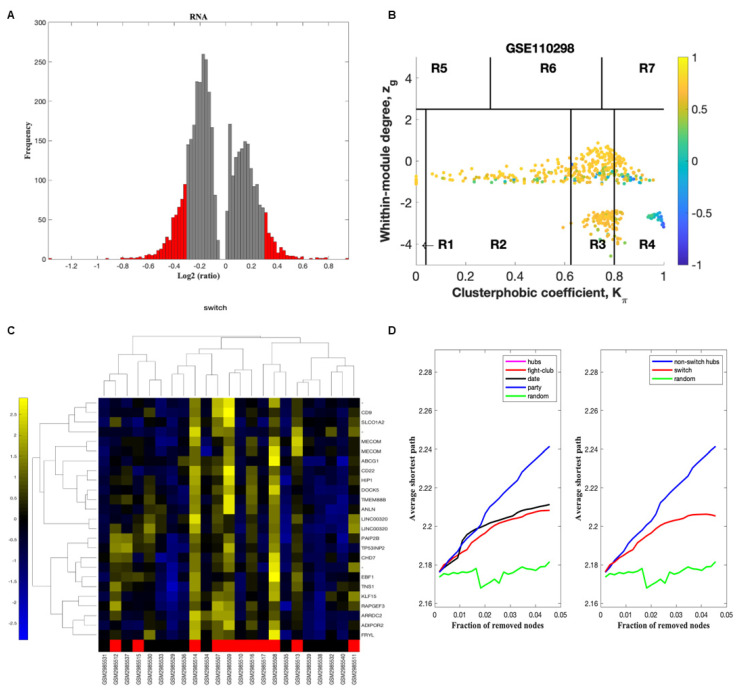
Swim analysis. SWIM analysis of post-mortem hippocampus brain region from cognitively intact, aged subjects engaged in various physical activity levels (GSE110298). (**A**) Distribution of log2 fold change values where the red bars are selected for further analysis. (**B**) Heat Cartography Map with nodes colored by their average Pearson Correlation Coefficient. Region R4 represents the switch genes. (**C**) Dendrogram and heat map for switch genes. The red markers indicate samples from subjects who engaged in low physical activity. (**D**) Robustness of the correlation network.

The gene expression dataset GSE110298 was exported into SWIM to identify key switch genes associated with physical activity in the hippocampus of aged cognitively intact subjects [11,14]. The dataset was analyzed using three comparisons: low vs. moderate, low vs. high physical activity, and high vs. moderate physical activity. In the first step, genes are retained (red bars) or eliminated (gray bars) using a cut-off of 1.5 or higher (Figure 2A).

We next identified the correlation communities based on the average Pearson correlation coefficient (Figure 2B). The nodes with a negative correlation value with their interaction partner, known as fight club hubs, are shown in R4 in blue (Figure 2B). Two parameters identify the plane: *Zg* (within-module degree) and *Kπ* (clusterphobic coefficient), and it is divided into seven regions, each defining a specific node role (R1–R7). High *Zg* values correspond to hubs nodes within their module (local hubs), whereas low *Zg* values correspond to nodes with few connections within their module (non-hubs within their communities, but they could be hubs in the network). Each node is colored according to its average Pearson Correlation coefficient (APCC) value. Yellow nodes are party and date hubs, positively correlated in expression with their interaction partners. Blue nodes are the fight-club hubs, with an average negative correlation in expression with their interaction partners. Blue nodes falling in the region R4 are the switch genes characterized by low *Zg* and high *Kπ* values and are connected mainly outside their module.

A dendrogram and heat map of the expression of the switch genes is shown in Figure 2C. The expression profiles of switch genes (including protein-coding and non-coding RNAs) are clustered according to rows (switch genes) and columns (samples) of the switch genes expression data (biclustering). The colors represent different expression levels that increase from blue to yellow. The samples depicted in red are from subjects with low physical activity. Most of the switch genes identified in individuals with low physical activity were upregulated (shown in yellow), whereas those with high physical activity were downregulated (shown in blue) (Figure 2C).

As shown in Figure 2D, fight-club hubs differ from date and party hubs, and switch genes are significantly different from random, confirming the analysis’s robustness. The x-axis represents the cumulative fraction of removed nodes, while the y-axis represents the average shortest path. The shortest path between two nodes is the minimum number of consecutive edges connecting them. Each curve corresponds to the variation of the average shortest path of the correlation network as a function of removing nodes specified by the colors of each line.

SWIM analysis identified 28 switch genes associated with different levels of physical activity in the hippocampus. Analysis of gene expression profiles from subjects engaging in low vs. high physical activity revealed 20 switch genes, referred to as low physical activity switch genes. Similarly, analysis of gene expression data from subjects engaging in high vs. moderate physical activity identified eight switch genes, referred to as high physical activity switch genes. No switch genes were identified in the low versus moderate activity analysis. The list of switch genes is presented in Table S2.

### 2.3. Biological and Functional Analysis of Switch Genes

Network and pathway analysis of switch genes was performed using NetworkAnalyst [15]. The lists of low and high physical activity switch genes were analyzed separately. Low physical activity switch genes associated with antigen activation of B cell receptor, nerve growth factor signaling, adaptive immunity, regulation of signaling by CBL, co-stimulation by the CD28 family, signaling by interleukins, interleukin 2 signaling, cytokine signaling, EGFR signaling, platelet aggregation, cell-surface interactions at the vascular wall, and hemostasis. High physical activity switch genes associated with telomerase maintenance, processing of DNA double-strand break, EGFR downregulation, homologous DNA repair, signaling by EFGR, chromosome maintenance, nuclear receptor transcription pathway, homologous recombination repair, double-strand break repair, extension of telomeres, and nucleotide excision repair (Table S3).

Transcription factor analysis of low and high physical activity switch genes identified 34 and 7 transcription factors, respectively (Table S4). The most significant transcription factors regulating the low and high physical activity switch genes according to degree and betweenness centrality were NRF1 and CREM, respectively.

### 2.4. Physical Activity Switch Genes Associated with Neurodegeneration

We investigated whether physical activity-related switch genes were involved in cognition or neurodegeneration, given the linkage between physical activity and brain health. To this end, we compared the results from this study to our previous analyses of switch genes in AD, FTD, and ALS [11,12,16]. Four physical activity-related switch genes, MAP4, CD9, SLCO1A2, and HIP1, were identified as switch genes in AD and ALS [11,16].

Functional associations were explored further using the HUGO database (Table S5). Several switch genes, including PAIP2B, RAPGEF3, ARRDC2, ABCG1, HIP1, and TP53INP2 associated with protein processing pathways, whereas others, including KLF15, ADIPOR2, SLCO1A2, and ABCG1 associated with metabolic processing of glucose, lipids, cholesterol, and bile salts. Other relevant functional associations were observed for switch genes LPAR1, CHD7, and FRYL, associated with neurogenesis and neuron projection development.

### 2.5. Gene Expression Correlation between Physical Activity and Neurodegenerative Diseases

Differential gene expression and correlation analyses of GSE110298 were performed using BSCE. Transcriptomic data from subjects engaged in high and moderate physical activity were compared to those engaged in low physical activity using the meta-analysis and correlation engine tool in BSCE. This tool uses a non-parametric ranking approach. Each gene is given a ranking score based on the statistical significance and consistency of the gene across the queried biosets. A numerical score is assigned to each gene, from highest to lowest significance (100–1) in each dataset. The genes are then ranked by specificity (i.e., significance and consistency across the different datasets). This analysis identified 4636 differentially expressed genes. The gene with the highest score was amyloid beta (A4) precursor protein-binding family B member 2 (APBB2). APBB2 was downregulated in both males and females who engaged in high and moderate physical activity compared to low physical activity. The list of differentially expressed genes with their scores is provided in Table S6.

The molecular mechanisms by which physical activity may exert neuroprotection are not entirely understood. We studied the genetic overlap between physical activity and the most common neurodegenerative diseases to further explore this connection. Gene expression profiles from physically active subjects (GSE110298) were compared to those from neurodegenerative diseases. The number of differentially expressed genes and the direction of the fold changes were compared between any two datasets. Transcriptomic data from the brain regions commonly affected in AD, PD, FTD, and HD; the hippocampus, substantia nigra, frontal cortex, and caudate nucleus, respectively, were compared to those obtained from physically active subjects.

**Figure 3:**
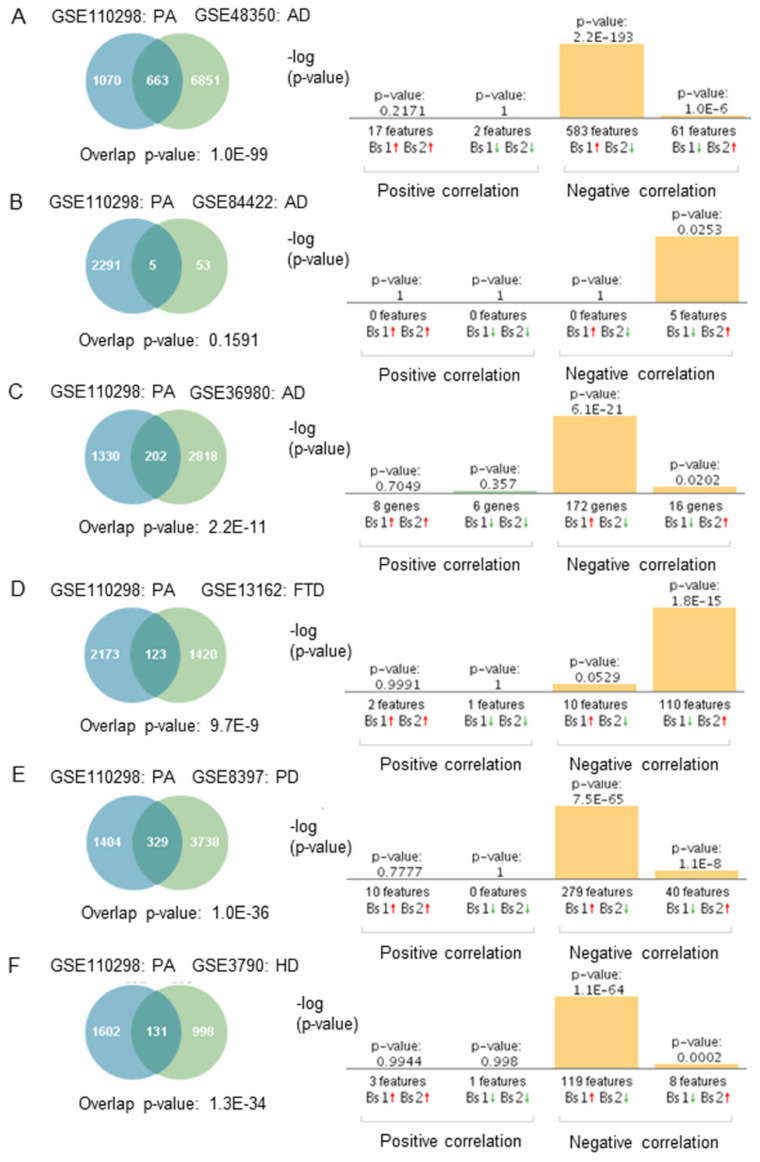
Genetic overlap and correlation analysis between subjects who engaged in high and moderate physical activity and patients with neurodegenerative diseases. Venn diagram and correlation analyses of shared genes between physically active (PA) subjects in GSE110298 and (**A**) AD GSE48350, (**B**) AD GSE84422, (**C**) AD GSE36980, (**D**) FTD-GRN (+) GSE13162, (**E**) PD GSE8397, (**F**) HD GSE3790. Vertical bars represent the significance of the overlap and the correlations between the datasets. Red and green arrows denote up and downregulation, respectively. *p*-value is expressed as the –log10 of the *p*-value. Statistical significances regarding the genetic overlap and the directionality of the fold changes were derived from the non-parametric ranking method used by Base Space Correlation Engine (Illumina, Inc., San Diego, CA, USA).

Gene expression profiles from subjects who engaged in moderate and high physical activity significantly overlapped with most of those from individuals with AD, PD, FTD, and HD (GSE48350, GSE84422, GSE8397, GS36980, GSE13162, GSE7621, GSE8397, GSE3790) (Figure 3A,C–F). The genetic overlap was insignificant in one array of AD patients (Figure 3B, *p* = 0.1591). Correlation analysis showed that gene expression profiles from physically active subjects correlated negatively with those from neurodegenerative diseases, AD, PD, FTD, and HD (Figure 3A–F). In the FTD dataset (GSE13162), a significant negative correlation was sustained in FTD-GRN (+) and FTD-GRN (−) in the hippocampus and frontal cortex brain regions. Correlations were observed in both males and females across all the datasets. Regarding the directionality of the fold changes, most of the overlapping genes were regulated in opposite directions. For example, genes upregulated in physically active subjects were downregulated in neurodegenerative diseases. The results from all the correlation analyses are presented in Table S7.

**Figure 4:**
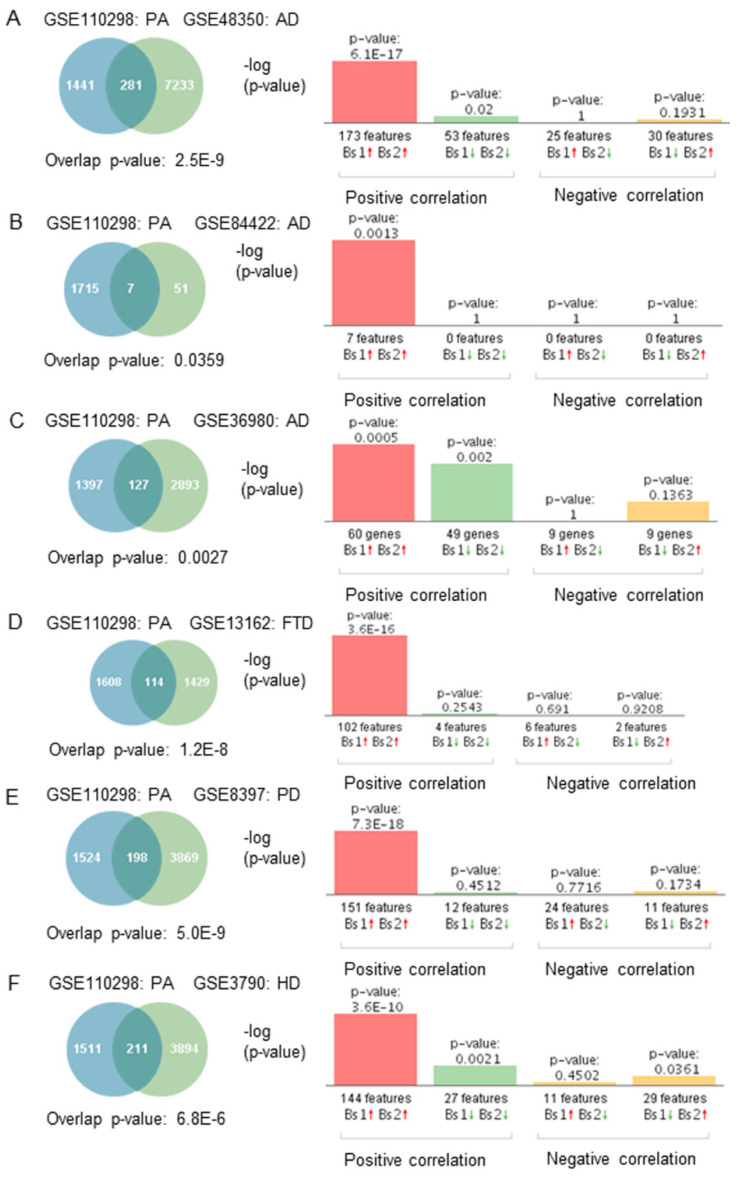
Genetic overlap and correlation analysis between subjects who engaged in low physical activity and patients with neurodegenerative diseases. Venn diagram and correlation analyses of shared genes between physically active (PA) subjects in GSE110298 and (**A**) AD GSE48350, (**B**) AD GSE84422, (**C**) AD GSE36980, (**D**) FTD-GRN(+) GSE13162, (**E**) PD GSE8397, (**F**) HD GSE3790. Vertical bars represent the significance of the overlap and the correlations between the datasets. Red and green arrows denote up and downregulation, respectively. *p*-value is expressed as the –log10 of the *p*-value. Statistical significances regarding the genetic overlap and the directionality of the fold changes were derived from the non-parametric ranking method used by Base Space Correlation Engine (Illumina, Inc., San Diego, CA, USA).

If gene expression patterns from subjects engaged in high/moderate physical activity correlated negatively with neurodegeneration, the opposite would be expected in subjects engaged in low physical activity. We compared gene expression profiles from subjects who engaged in low physical activity to those affected by neurodegenerative diseases to test this hypothesis. Correlation analysis showed that gene expression patterns from individuals who engaged in low physical activity correlated positively with those affected by neurodegenerative diseases (Figure 4A–F). Furthermore, most of the overlapping genes were regulated in the same direction. For example, genes upregulated in subjects engaged in low physical activity levels were upregulated in the brain of subjects with neurodegenerative diseases (Figure 4A–F).

**Figure 5:**
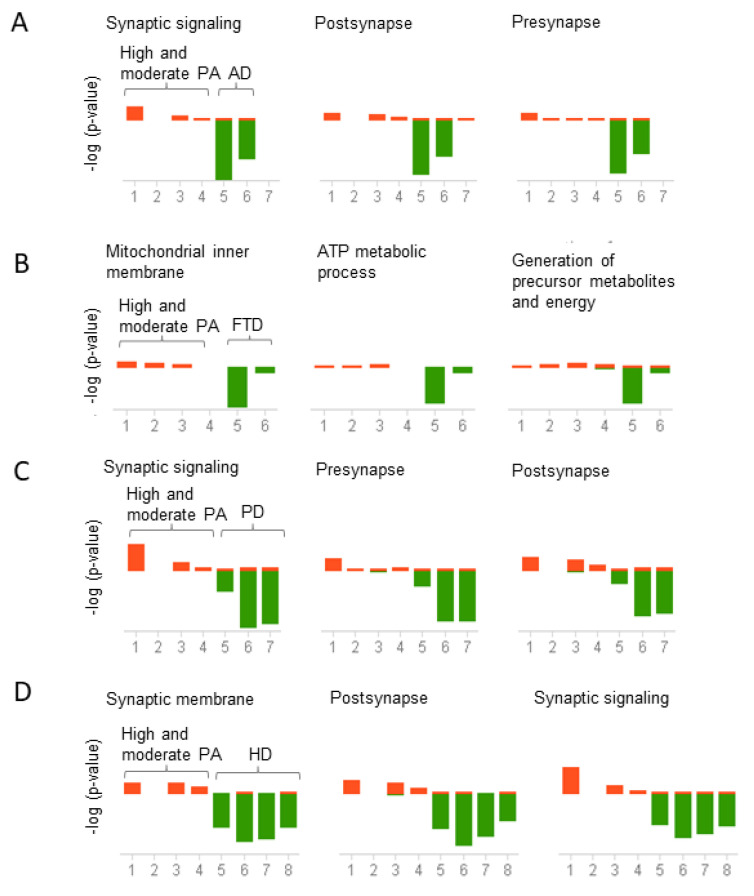
Correlation analysis of pathways shared between physical activity and neurodegenerative diseases. Genes upregulated in physical activity (PA) and downregulated in neurodegenerative diseases were analyzed using the meta-analysis and correlation in BSCE. The analysis was performed for each neurodegenerative disease separately. Gene ontology (GO) association terms were used for functional analysis. GO groups were filtered using a minimum size of 10 genes and a maximum of 500 genes per group. Each correlated biogroup has an associated score matrix. The height of each vertical bar represents the score of the correlation between the queried dataset and the biogroup (GO group). The orange bar above the midline represents the overlap, or enrichment, between the dataset and the upregulated genes in the dataset. Likewise, the green bar below the midline represents the overlap, or enrichment, between the dataset and the downregulated genes in the dataset. The absence of a colored bar means that the correlation is insignificant. The datasets analyzed are represented on the x-axis. High and moderate physical activity datasets (1–4) compared to (**A**) Alzheimer’s disease (AD) datasets (5–7), (**B**) Frontotemporal dementia (FTD) datasets (5–6), (**C**) Parkinson’s disease (PD) datasets (5–7), (**D**) Huntington’s disease (HD) datasets (5 = HD grade 1, 6 = HD grade 2, 7 = HD grade 3, 8 = HD grade 4).

To investigate the biological and molecular pathways altered by physical activity, we conducted a gene ontology analysis in BSCE. Given the negative correlation between physical activity across all the neurodegenerative diseases studied herein, we analyzed the genes differentially expressed in opposite directions between physical activity and neurodegenerative diseases. For instance, functional analysis was performed on the group of upregulated genes in subjects who engaged in high and moderate physical activity and downregulated in the different neurodegenerative diseases and vice versa. Genes upregulated in high and moderate physical activity and downregulated in AD, PD, and HD were prominently enriched in synaptic signaling, postsynapse, and presynapse (Figure 5A,C,D). In contrast, downregulated genes in FTD are associated with mitochondrial inner membrane, ATP metabolic process, and precursor metabolites and energy (Figure 5B).

**Figure 6:**
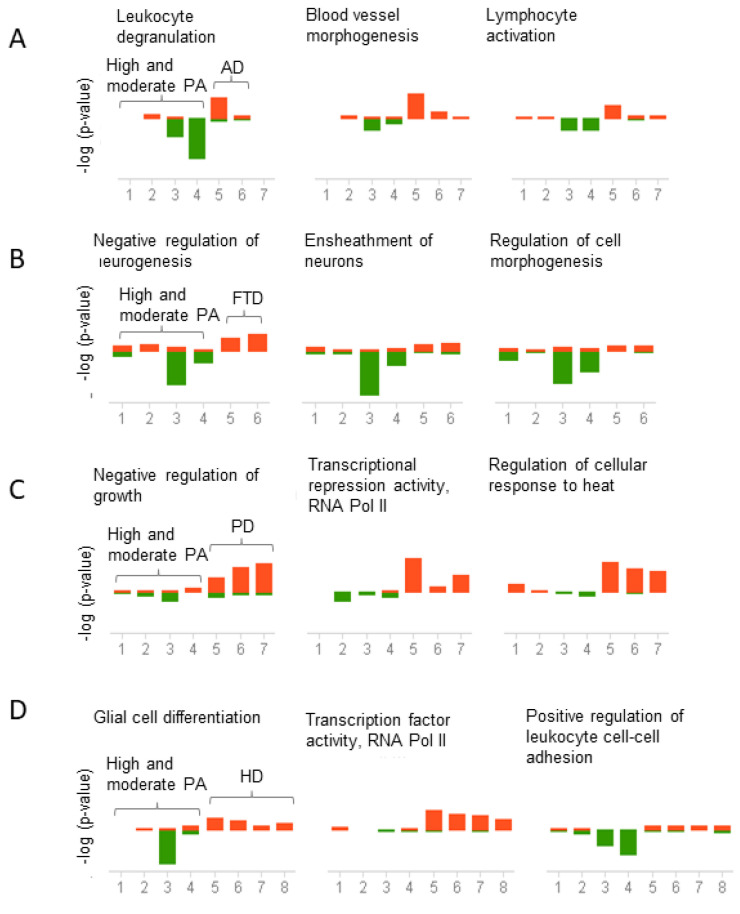
Correlation analysis of pathways shared between physical activity and neurodegenerative diseases. Genes downregulated in physical activity (PA) and upregulated in neurodegenerative diseases were analyzed using the meta-analysis and correlation in BSCE. The analysis was performed for each neurodegenerative disease separately. Gene ontology (GO) association terms were used for functional analysis. GO groups were filtered using a minimum size of 10 genes and a maximum of 500 genes per group. Each correlated biogroup has an associated score matrix. The height of each vertical bar represents the score of the correlation between the queried dataset and the biogroup (GO group). The orange bar above the midline represents the overlap, or enrichment, between the dataset and the upregulated genes in the dataset. Likewise, the green bar below the midline represents the overlap, or enrichment, between the dataset and the downregulated genes in the dataset. The absence of a colored bar means that the correlation is insignificant. The datasets analyzed are represented on the x-axis. High and moderate physical activity datasets (1–4) compared to (**A**) Alzheimer’s disease (AD) datasets (5–7), (**B**) Frontotemporal dementia (FTD) datasets (5–6), (**C**) Parkinson’s disease (PD) datasets (5–7), (**D**)Huntington’s disease (HD) datasets (5 = HD grade 1, 6 = HD grade 2, 7 = HD grade 3, 8 = HD grade 4).

Conversely, genes downregulated in high and moderate physical activity and upregulated in AD were enriched in leukocyte degranulation, blood vessel morphogenesis, and lymphocyte activation (Figure 6A). In FTD, genes were enriched in the negative regulation of neurogenesis, ensheathment of neurons, and regulation of cell morphogenesis (Figure 6B). In PD, genes were enriched in negative regulation of growth, transcriptional repressor activity, and regulation of cellular response to heat (Figure 6C). Finally, in HD, genes were enriched in glial cell differentiation, transcription factor activity, and positive regulation of leukocyte cell-cell adhesion (Figure 6D). The top three pathways are presented in Figure 5 and Figure 6, but the complete list of functional associations is provided in Tables S8–S15.

## 3. Discussion

### 3.1. Switch Genes Associated with Physical Activity

Here we used bioinformatic analyses to identify genes responsible for dramatic transcriptional changes occurring in the brain of healthy aging individuals who engaged in different physical activity levels. Co-expression network analysis identified 28 switch genes associated with varying physical activity levels in the hippocampus of aged cognitively intact individuals.

Functional analysis of low physical activity switch genes identified pathways involved in immune system mechanisms. It is well documented that a sedentary lifestyle is associated with an increased risk of metabolic syndrome and systemic inflammation [17,18]. In this regard, inflammation plays a central role in the pathogenesis of neurodegenerative diseases [19]. Conversely, switch genes obtained from subjects engaged in high physical activity were predominantly associated with DNA repair mechanisms and telomerase maintenance. Telomerase maintenance positively influences longevity [20], and telomeric dysfunction is associated with developing a wide range of diseases, including cancer, metabolic disorders, and neurodegeneration [21]. Increased DNA double-strand breaks and altered levels of DNA repair proteins have been reported in the hippocampus of AD subjects [22] and neuronal and glial cells in the early stages of the disease [23]. Likewise, DNA strand breaks and damage response pathways may contribute to disease pathology in PD [24,25]. These findings suggest that high physical activity influences the transcriptional regulation of genes involved in DNA damage, possibly preventing neurodegeneration. In contrast, low physical activity induces transcription of gene mediators of inflammation.

### 3.2. Transcription Factors Regulation of Switch Genes

Transcription factor analysis of low physical activity switch genes identified NRF1 as the most highly ranked transcription factor. NRF1 plays a crucial role in controlling the proteasome function [26]. Aberrant protein degradation and accumulation in brain tissues is central to the pathogenesis of AD, PD, and HD. Several switch genes, including PAIP2B, RAPGEF3, ARRDC2, ABCG1, HIP1, and TP53INP2 associated with protein processing pathways, further reinforcing the involvement of physical activity in proteasome function. These findings suggest that low physical activity may impair proteasome function leading to the accumulation of unwanted misfolded proteins.

Transcription factor analysis of high physical activity switch genes identified CREM as the most highly ranked transcription factor. CREM transcriptional activity controls cytokine expression of T lymphocyte-specific target genes, including IL2 and IL17 cytokines [27]. Interestingly, IL17 has been the focus of recent investigations, suggesting it may be a central cytokine in developing neurodegenerative diseases [28]. IL17 has been reported to activate microglia and exacerbate neuroinflammation and neurodegeneration in a rodent PD model [29]. Microglial activation has emerged as a critical mechanism in the pathogenesis of neurodegenerative diseases [30,31]. Recent evidence suggests that lower microglia activation is linked to age-related brain health in humans [9]. Another switch gene, CD22, is a negative regulator of phagocytosis, and it is upregulated in the aged microglia [32]. Strikingly, antibody blockage of CD22 mediated the clearance of amyloid-β oligomers and α-synuclein fibers, and improved cognitive function in mice [32]. These findings suggest that high physical activity reprograms the brain towards a lower inflammatory state and that microglial activation and CD22 may play a role in neurodegeneration.

### 3.3. Switch Genes Associated with Neurodegeneration

Several switch genes obtained from the low physical activity subjects are involved in neurodegeneration. For instance, EBF1 is responsible for the downregulation of FAM3C, an endogenous suppressor of β amyloid production in AD [33]. RAFGEF3, also known as EPAC1, is involved in a protective role in suppressing the nucleocytoplasmic transport associated with neurodegeneration in ALS and FTD [34]. TP53INP2 is highly expressed in sympathetic neuron axons and regulates axon growth by enhancing NGF-TrkA signaling [35], a pathway implicated mainly in neurodegenerative diseases [36,37].

A sedentary lifestyle can negatively impact glucose, lipid, and cholesterol metabolism, leading to metabolic syndrome and neurodegenerative diseases. Several low physical activity switch genes are involved in lipid, cholesterol, and glucose metabolism. For instance, KLF15 is an important regulator of glucose and lipid metabolism [38,39]. Mice with skeletal muscle deletion of KLF15 showed insulin resistance, glucose intolerance, and increased lipid deposition [38]. Insulin resistance and dysregulation of lipid homeostasis have been extensively implicated in the pathogenesis of AD and PD [40,41,42,43,44]. Interestingly, a high protein diet, a lifestyle modification beneficial for many diseases, controls the transcription of genes involved in glucose and lipid metabolism via KLF15 [45]. Another switch gene associated with low physical activity, DOCK5, is associated with severe obesity in children and adults [46,47]. Deletion of DOCK5 in mice promoted obesity, insulin resistance, and altered glucose metabolism via activation of the mTOR/S6K1 pathway [48]. Interestingly, DOCK5 is overexpressed in the substantia nigra of PD patients [49], and diabetes is associated with the development of PD [40]. Similarly, ADIPOR2 is implicated in obesity. For example, overexpression of miR-126b-5p promoted obesity in mice by directly targeting Adipor2 [50].

Another switch gene associated with low physical activity, ABCG1, is a cholesterol transporter that helps prevent the formation of high-density lipoprotein and atherosclerosis [51]. Interestingly, ABCG1 decreases γ-secretase activity, thus preventing the accumulation of amyloid β [52]. Targeting ABCG1 has been suggested to reduce the risk of atherosclerosis and AD [53]. Low physical activity can provoke drastic transcriptional changes in genes important in regulating glucose, lipids, and cholesterol metabolism, ultimately leading to neurodegeneration.

Other switch genes, including LPAR1, MAP4, and MAP4k4, identified from the high physical activity transcriptomic profiles, have been shown to play important functions in the central nervous system. For example, LPAR1 is highly expressed in the brain and regulates cellular proliferation, migration, and apoptosis [54]. Moreover, dysregulation of LPAR1 has been associated with neurodevelopmental disorders, demyelination diseases, and neuropathic pain [54]. In addition, LPAR1 is an intrinsic modulator of axon growth in corticospinal motor neurons after injury [55], and it may be involved in microglial activation after CNS injury [56] via MAPK signaling [57].

Several switch genes associated with physical activity have been identified as switch genes in our previous studies on AD and ALS. For example, switch genes CD9 and MAP4, associated with low and high physical activity, respectively, were also identified as switch genes in the hippocampus of AD subjects [11]. Additionally, HIP1 and SLCO1A2 were also identified as switch genes in spinal motor neurons of ALS patients [16]. CD9 is upregulated in the mouse and human brains infected with transmissible spongiform encephalopathy [58]. In addition, CD9 has been reported as a biomarker of sodium cyanide exposure [59], a hazardous chemical linked to neurodegeneration [60], and PD [61]. HIP1 is associated with Huntington’s disease and neuronal cell death through the intrinsic apoptotic pathway [62]. A genetic variant near SLCO1A2 is related to cortical beta-amyloid burden and cognitive dysfunction [63]. In addition to AD, a SLCO1A2 genetic variant is associated with progressive supranuclear palsy [64]. The involvement of switch genes in neurodegenerative diseases reinforces the notion that physical activity is a lifestyle modification that promotes brain health and may prevent neurodegeneration.

### 3.4. Physical Activity Opposes Transcriptional Changes Associated with Neurodegeneration

In addition to switch genes, we investigated transcriptional changes associated with physical activity by traditional differential gene expression analysis. Using the meta-analysis and correlation engine tool in BSCE, we compared the transcriptomic profiles of subjects who engaged in high and moderate physical activity to those from low physical activity. APBB2 was identified as the most highly ranked gene. APBB2 was downregulated in both males and females who engaged in high and moderate physical activity compared to low physical activity. This finding is intriguing given the critical role that APBB2 plays in the pathogenesis of AD. Overexpression of APBB2 promotes the accumulation of β-amyloid, and some of its genetic variants are associated with severe cognitive impairment and late-onset AD [65,66,67,68]. These findings suggest that high and moderate levels of physical activity alone can reduce the expression of molecular drivers of neurodegeneration.

We next investigated how gene expression patterns from physically active subjects overlap with those from neurodegenerative diseases. Correlation analysis revealed that gene expression profiles from individuals who engaged in high and moderate physical activity negatively correlated with those with AD, PD, FTD, and HD. Furthermore, these correlation trends were sustained in sporadic FTD, genetic forms of FTD, and HD, an autosomal dominant disorder. Conversely, gene expression patterns from individuals with low physical activity correlated positively with those from neurodegenerative diseases. These findings suggest that high and moderate physical activity influences the transcription of genes that oppose the neurodegenerative processes. In contrast, low physical activity promotes the expression of those genes that trigger neurodegeneration, even in genetic forms of the disease.

Functional analysis revealed that genes involved in synaptic signaling were prominently downregulated in AD, PD, and HD and upregulated by high and moderate physical activity. Synapse dysfunction is associated with early stages of neurodegeneration and symptoms in PD and HD [69,70,71]. Synapse-related genes are extensively downregulated in multiple brain regions in both aging and AD [72]. Furthermore, greater physical activity is associated with higher levels of synaptic integrity markers, including synaptophysin, synaptotagmin-1, vesicle-associated membrane proteins, syntaxin, and complexin-I, in the brain of older adults [73].

Interestingly, in FTD, the most significant pathways that inversely correlated with physical activity were related to cellular energy metabolism, including mitochondrial inner membrane, ATP metabolism, and generation of energy precursors. This is not surprising since C9orf72, a significant risk factor for ALS and FTD is a mitochondrial inner membrane protein that plays a critical role in regulating cellular energy homeostasis [74]. Further, physical exercise improved energy metabolism via the upregulation of glucose transporters and metabolic enzymes in the skeletal muscle of ALS mice [75]. Downregulation of the mitochondrial electron transport chain has been reported in human transcriptomic datasets from ALS-FTD patients [76]. These findings suggest that physical activity may confer neuroprotection through different pathways in neurodegenerative diseases. For instance, physical activity may counteract the transcriptional changes associated with synaptic loss in AD, PD, and HD. In contrast, in FTD, physical activity may restore mitochondrial function and metabolic energy precursors.

Other pathways that demonstrated inverse associations between physical activity and neurodegeneration were related to inflammation, neurogenesis, regulation of growth, and differentiation. For example, immune-associated pathways, including leukocyte degranulation and lymphocyte activation, were prominently upregulated in AD and downregulated in high and moderate physical activity. Negative regulation of neurogenesis and myelination were primarily upregulated in FTD and downregulated in physical activity. In PD, negative growth regulation and transcriptional repressor activity were predominantly upregulated compared to physical activity. Finally, in HD, glial cell differentiation and transcription factor activity were consistently upregulated across the different stages of HD progression.

In support of these findings, a transcriptomic meta-analysis of human datasets from different brain regions affected in AD, ALS, and FTD identified a common signature characterized by an upregulation of innate immunity, transcription, and RNA processing genes, and downregulation of mitochondrial oxidative phosphorylation, and ubiquitin-proteosome pathways [76]. Despite differences in neuropathology and brain regions affected, we showed that physical activity might alter these pathways in different neurodegenerative diseases.

Some limitations of this study are noteworthy. The observations in this study are entirely based on bioinformatic methods and correlational associations. Therefore, a causal relationship between physical activity and neuroprotection cannot be established. Additionally, differences in different populations’ genetic and cultural backgrounds are potential confounding variables. Future prospective studies of physical activity on subjects at risk of neurodegenerative diseases would help confirm these associations. Studies investigating multidomain lifestyle interventions, including diet, physical activity, cognitive training, sleep improvement, meditation and mindfulness training, social support, and reduced exposure to toxins in individuals at risk for dementia or neurodegeneration will be crucial to understanding better how lifestyle factors affect the brain on a cellular and molecular level.

## 4. Materials and Methods

### 4.1. Microarray Dataset Selection

We searched the GEO database (https://www.ncbi.nlm.nih.gov/gds (accessed on 2 January 2022)) and BaseSpace Correlation Engine (BSCE, Illumina, Inc., San Diego, CA, United States) for brain transcriptomic studies using the search terms “homo sapiens”, “human”, “physical activity”, “exercise”, “brain”. In addition, we searched microarrays from neurodegenerative diseases using the terms “homo sapiens”, “Alzheimer’s disease”, “Parkinson’s disease”, “Huntington’s disease”, “Frontotemporal dementia”, “brain”, “hippocampus”, “frontal cortex”, “substantia nigra”, “caudate nucleus”, “motor cortex”. For all the arrays, inclusion criteria were: (1) human microarrays from relevant brain tissues in AD, FTD, PD, and HD. Exclusion criteria were: (1) animal models, (2) cellular and patient-derived stem cells, and (3) incompatible technologies. One dataset for physical activity met our inclusion/exclusion criteria, GSE110298 containing transcriptomic data from elderly cognitively normal individuals and clinically well-defined patients. In this study, exercise and non-exercise physical activity was measured longitudinally on an annual basis. Seven microarrays from neurodegenerative diseases met our inclusion/exclusion criteria.

These microarrays were analyzed further using the meta-analysis and correlation analysis tools in BSCE. Although BSCE corrects for differences in array platforms, we selected those arrays using the same microarray platform to ensure consistency and data comparability across the arrays. All the chosen arrays used the Affymetrix Human Genome platform. The microarrays analyzed in this study are described in Table S1.

### 4.2. Description of Microarrays

Tissue samples in dataset GSE110298 were obtained post-mortem from the mid-hippocampus region from aged, cognitively intact, and healthy individuals enrolled in the Rush Memory and Aging Program (MAP). Physical activity was continuously monitored with actigraphy and categorized as light, moderate, or high physical activity. The study participants from MAP were described as healthy, with preserved cognition, older (aged 76–100 years), and without a known history of dementia or neurodegenerative disease. Study participants were recruited from northern Illinois. Cognitive performance was assessed using a battery of 21 cognitive tests. Lifestyle factors and comorbidities were also collected. History of cardiovascular risk factors, cardiovascular disease, musculoskeletal pain, medication, alcohol, and tobacco use was also documented. Most of the comorbid disease analyses were assessed by self-report.

Physical activity was continuously monitored by actigraphy using a waterproof wristwatch-size placed on the non-dominant wrist of each participant and worn 24 h a day. The actigraphy measured periods of activity and rest. The average physical activity was calculated as the average across days of the total physical activity counts recorded. The average range of daily physical activity was 0.3–5.6 × 10^5^ activity counts per day. This data was converted to approximate metabolic equivalents (METs). Briefly, 1.0 × 10^5^ was equivalent to 20 min/day of moderate-high activity or 1.5 METs-h of activity per day. Using these metrics, physical activity was categorized into 3 levels: low activity: <1.05 × 10^5^ activity counts/day, moderate activity: 1.06 × 10^5^ × 2.5 × 10^5^ activity counts/day, high activity: >2.5 × 10^5^ activity counts/day. These thresholds for the different physical activity levels were defined previously in [7,10]. Detailed information about study participants, clinical assessments, and evaluation of physical activity can be found elsewhere [10].

We identified seven brain transcriptomic studies encompassing the broad spectrum of neurodegenerative diseases, including AD, PD, FTD, and HD. GSE48350 included AD cases from 7 brain banks from the National Institute on Aging Alzheimer’s disease. This dataset included 18 samples from the hippocampus of AD cases (84.2 ± 6.8 years) and 43 healthy controls. Males and females were similarly represented. Each group was balanced for the APOE genotype. AD cases showed a progressive cognitive decline. Mini-mental status examination was 18.32 ± 9.19, Braak stage II-VI (average stage IV). Neuropathological diagnosis was based on the Consortium to Establish a Registry for Alzheimer’s Disease (CERAD) and National Institute of Aging and Reagan Institute criteria. Controls were cognitively intact, with MMSE scores ranging from 26 to 30 (28.35 ± 1.57). Exclusion criteria for both AD and controls included evidence of alcoholism, psychiatric illness, major depression, head trauma, coma, brain cancer, and a history of cerebrovascular disease. Detailed clinical and demographic information can be found elsewhere in [72].

A subset of samples from GSE84422 was analyzed and included 18 hippocampus samples from individuals with a definitive diagnosis of AD and 11 age- and sex-matched healthy controls from the Mount Sinai Medical Center Brain Bank. Detailed information about the clinical and neuropathological assessments, including Braak stage, CERAD ratings, plaque, and NFT densities, can be found elsewhere [77]. GSE36980 dataset included hippocampus samples from 7 AD subjects (93.06 ± 6.14 years) and 10 HC (78.30 ± 10.46). Assessment of AD neuropathology was performed according to CERAD guidelines and the Braak stage. GSE13162 microarray contained hippocampus transcriptomic data from 5 subjects with FTD with progranulin gene mutations (FTD-GRN) and 8 FTD without progranulin mutations (FTD-GRN (−)) and two age- and sex-matched HC. The median ages (interquartile range) for FTD-GRN (+), FTD-GRN (−), and HC were 71, 64, and 67, respectively. More information about the study participants can be found in [78]. GSE7621 dataset contained transcriptomic data from the substantia nigra of 16 PD patients (75.1 ± 7.81 years) and 9 HC (77.9 ± 13.09). Post-mortem brain tissue was obtained from the brain bank at the University of Miami. Clinical and pathological assessments of PD subjects were based on the UK PD Society Brain Bank diagnostic criteria and Hoehn and Yahr scale. Details about clinical and demographic information were published in [79]. GSE8397 included transcriptomic data from the lateral and medial substantia nigra from PD patients (80 ± 5.7 years) and HC (70.6 ± 12.5 years). Brain samples were obtained from the UK Parkinson’s Disease Society Tissue Bank at Imperial College London. More information about study participants is published elsewhere [80]. GSE3790 included brain transcriptomic data from the caudate nucleus of 34 HD patients (median age 61.5 years) and 32 HC (median 62.5 years). HD subjects were analyzed based on the presence or absence of symptoms and Vonsattel grade of HD pathology (grade 0–4). All the HD subjects were in the pathological range of more than 35 CAG repeats. Clinical and demographic information can be found elsewhere [81].

### 4.3. Swim Analysis to Identify Switch Genes

Raw gene expression data from dataset GSE110298 was imported into SWIM [82]. The SWIM algorithm was described in detail in [14,82]. Briefly, genes with no or low expression were removed in the preprocessing stage. The fold changes were set for each array in the filtering step, and genes that were not significantly expressed between cases compared to controls were removed. The False Discovery Rate method (FDR) was used for multiple test corrections. Pearson correlation analysis was performed to build a co-expression network of genes differentially expressed between cases and controls. The k-means algorithm was used to identify communities within the network, as previously demonstrated [14]. SWIM uses a Scree plot to determine the number of clusters, and the clusters with the lowest number of sums of the square error (SSE) values among the replicates are designated as the number of clusters. A heat cartography map is built using a clusterphobic coefficient *Kπ* and the global-within module degree Zg. The coefficient *Kπ* measures the external and internal node connections, whereas *Zg* measures the extent each node is connected to others in its community. A node is considered a hub when Zg > 5. The average Pearson correlation coefficient (APCC) between the expression profile of each node and its nearest neighbors is used to build the heat cartography map. Using the APCC, three types of hubs are defined; date hubs that show low positive co-expression with their partners (low APCC), party hubs that show high positive co-expression (high APCC), and nodes that have negative APCC values are called fight-club hubs. In the final step, switch genes are identified, a subset of the fight-club hubs interacting outside their community. Switch genes are defined as not being a hub in their cluster (low *Zg* < 2.5), having many links outside their cluster (*Kπ* > 0.8, when *Kπ* is close to 1, most of its links are external to its module), and having a negative average weight of incident links (APCC < 0). Switch genes interact outside their community, are not in local hubs, and are mainly anti-correlated with their interaction partners [14].

### 4.4. Pathway Analysis

For network and pathway analysis, official gene symbols for the switch genes were imported into NetworkAnalyst, https://www.networkanalyst.ca/ (accessed on 18 February 2022). The minimum connected network was selected for further pathway analysis. Tissue-specific networks were built using the hippocampus protein–protein interaction database in NetworkAnalyst. Tissue-specific protein–protein interaction data from the DifferentialNet database showed the differential protein–protein interactions across human tissues. Data derived from Reactome were used for pathway selection. Shared pathways were identified using a Venn diagram analysis. Hugo Gene Nomenclature Committee (HUGO) database was used for gene ontology analysis (https://www.genenames.org/ (accessed on 18 February 2022).

### 4.5. Gene-Transcription Factors Interaction Analysis

Gene-transcription factors interactome was performed in NetworkAnalyst. Switch genes obtained from subjects engaging in low and high physical activity were analyzed separately. Transcription factor and gene target data were derived from the Encyclopedia of DNA Elements (ENCODE). ENCODE uses the BETA Minus Algorithm in which only peak intensity signal <500 and the predicted regulatory potential score <1 are used. Transcription factors were ranked according to network topology measurements, including degree and betweenness centrality.

### 4.6. Gene Expression and Correlation Analyses

Gene expression and correlation analyses were performed using the curated database BSCE. The dataset GSE110298 was analyzed using the meta-analysis and correlation tool in BSCE. Differentially expressed genes were analyzed between cases and controls. The gene expression profiles of subjects who engaged in physical activity (GSE110298) were compared to gene expression profiles from subjects with neurodegenerative diseases using the correlation tool. The genetic overlap between GSE110298 and the other datasets were analyzed as previously [42]. BSCE uses a “Running Fisher” algorithm to compute the overlapping *p*-values between different gene expression datasets [83]. BSCE replaces any negative values with the smallest positive number in the dataset to analyze differentially expressed genes. Genes below the 20th percentile of the combined normalized signal intensities were removed. BSCE uses a normalized ranking approach, which enables the comparison between different datasets independently of the absolute fold changes. The scoring and ranking of a gene are calculated according to the activity of each gene in each dataset and the number of datasets in which the gene is differentially expressed. Ranks are normalized to eliminate bias owing to varying platform sizes. However, we included only studies that employed the same platform in this study, thus removing this variable. Only genes with a *p*-value of 0.05 or less and an absolute fold-change of 1.2 or greater were regarded as significant.

For the correlation analysis, the number of differentially expressed genes and the direction of the fold changes were compared between any two datasets. Gene expression patterns from the brain regions most commonly affected in AD, PD, FTD, and HD—the hippocampus, substantia nigra, frontal cortex, and caudate nucleus, respectively—were compared to those obtained from subjects engaged in physical activity (GSE110298).

## Data Availability

The original contributions presented in the study are included in the article/Supplementary Material, further inquiries can be directed to the corresponding author.

## Supplementary Materials

The following supporting information can be downloaded at: https://www.mdpi.com/article/10.3390/ijms23116223/s1.
