# Supplementary material for: Physical Activity Rewires the Human Brain against Neurodegeneration
Source: Int J Mol Sci. 2022 Jun 2;23(11):6223. doi: 10.3390/ijms23116223 (PMC9181322; doi:10.3390/ijms23116223)
Supplement: Supplementary file 1 [file ijms-23-06223-s001.zip › Table S1.pdf]

Table S1. Microarrays analyzed in this study.

**Accession number**	**Brain region**	**Description of samples**	**Platform**
GSE110298	hippocampus	Physical activity level<br>Low: 11<br>Moderate: 11<br>High: 12	Affymetrix Human Genome U133 Plus 2.0 Array
GSE48350	hippocampus	AD: 19<br>HC: 43	Affymetrix Human Genome U133 Plus 2.0 Array
GSE84422	hippocampus	AD:18<br>HC:11	Affymetrix Human Genome U133A Array
GSE36980	hippocampus	AD:7<br>HC:10	Affymetrix Human Gene 1.0 ST Array
GSE13162	Hippocampus<br>Frontal cortex	FTD-GRN(+): 5<br>FTD-GRN(-): 8<br>HC: 2	Affymetrix Human Genome U133A Array
GSE7621	Substantia nigra	PD:16<br>HC: 9	Affymetrix Human Genome U133 Plus 2.0 Array
GSE8397	Substantia nigra	PD:24<br>HC:23	Affymetrix Human Genome U133 Array Set HG-U133A
GSE3790	Caudate nucleus	HD:13<br>HC:32	Affymetrix Human Genome U133 Array Set HG-U133A

AD: Alzheimer's disease; HC: Healthy controls; FTD-GRN (+): Frontotemporal dementia with progranulin mutations; FTD-GRN (-): Frontotemporal dementia without progranulin mutations; PD: Parkinson's disease; HD: Huntington's disease
