# Supplementary material for: Physical Activity Rewires the Human Brain against Neurodegeneration
Source: Int J Mol Sci. 2022 Jun 2;23(11):6223. doi: 10.3390/ijms23116223 (PMC9181322; doi:10.3390/ijms23116223)
Supplement: Supplementary file 1 [file ijms-23-06223-s001.zip › Table S5.pdf]

Table S5. Gene ontology functional association of switch genes

Switch gene	Dataset	Gene ontology association
LPAR1 (Lysophosphatidic acid receptor 1)	High physical activity	Neurogenesis, regulation of metabolic process, positive regulation of MAPK, positive regulation of cytosolic Ca
SLC31A2 (Solute carrier family 31, member 2)	High physical activity	Copper ion transport and homeostasis
MAP4K4 (Mitogen activated protein kinase 4)	High physical activity	Focal adhesion, microtubule binding, apoptosis, cell migration, MAPK cascade, JNK cascade, Alzheimer's disease
PAIP2B (Poly(A) binding protein interacting protein 2B)	High and low physical activity	Regulation of translation
RAPGEF3 (Rap guanine nucleotide exchange factor 3)	Low physical activity	Guanyl-nucleotide exchange factor activity, Ras signal transduction, associative learning, protein acetylation, adaptive immune response
KLF15 (Kruppel like factor 15)	Low physical activity	Regulation of transcription, glial cell differentiation, positive regulation of glucose import, glucose homeostasis, response to insulin
TNS1(Tensin 1)	Low physical activity	Focal adhesion, fibroblast migration, RNA binding
DOCK5 (Dedicator of cytokinesis 5)	Low physical activity	Cell migration, guanyl-nucleotide exchange activity, epithelial and smooth muscle cell migration
HIP1 (Huntingtin interacting protein 1)	Low physical activity	Protein stabilization, presynapse, regulation of endocytosis, neurotransmitter receptor transport, ALS
ADIPOR2 (Adiponectin receptor 2)	Low physical activity	Adiponectin signaling, glucose homeostasis, vascular wound healing, lipid and fatty acid metabolism
MECOM (MDS1 and EVI1 complex locus)	Low physical activity	Regulation of cell cycle, programmed cell death, negative regulation of JNK, hematopoietic stem cell proliferation
TMEM88B (Transmembrane protein 88B)	Low physical activity	PDZ domain binding
ARRDC2 (Arrestin domain containing 2)	Low physical activity	Protein transport, protein binding
CHD7 (Chromodomain helicase DNA binding protein7)	Low physical activity	Chromatin binding, histone binding, cranial nerve development, heart morphogenesis, olfactory behavior, neurogenesis, cognition
CD9 (CD9 molecule)	Low physical activity	Glial cell migration, response to low density lipoprotein, cell

		adhesion, platelet activation, Alzheimer’s disease
ABCG1 (ATP binding cassette subfamily G member 1)	Low physical activity	Positive regulation of amyloid beta formation, cholesterol homeostasis, cholesterol esterification, response to HDL
EBF1 (EBF transcription factor 1)	Low physical activity	Regulation of transcription, DNA binding
CD22 (CD22 molecule)	Low physical activity	B cell activation, regulation of immune response, negative regulation of calcium signaling
FRYL (FRY like transcription coactivator)	Low physical activity	Neuron projection development, cell morphogenesis
ANLN (Anilin actin binding protein)	Low physical activity	Cell division, cell projection, cell cycle, hematopoietic cell differentiation
SLCO1A2 (Solute carrier organic anion transporter family member 1A2)	Low physical activity	Bile acid and bile salts transport, ALS
TP53INP2 (Tumor p53 nuclear protein 2)	Low physical activity	Autophagosome assembly, protein binding, ubiquitin dependent catabolic process, osteoblast differentiation

HDL: high density lipoprotein

ALS: Amyotrophic lateral sclerosis
